# Zinc Binding to NAP-Type Neuroprotective Peptides: Nuclear Magnetic Resonance Studies and Molecular Modeling

**DOI:** 10.3390/ph14101011

**Published:** 2021-10-01

**Authors:** Ancuta-Veronica Lupaescu, Cosmin Stefan Mocanu, Gabi Drochioiu, Catalina-Ionica Ciobanu

**Affiliations:** 1 Integrated Center for Research, Development and Innovation in Advanced Materials, Nanotechnologies and Distributed Systems for Fabrication and Control (MANSiD), Stefan cel Mare University of Suceava, 720229 Suceava, Romania; ancuta.lupaescu@usm.ro; 2 Faculty of Chemistry, Alexandru Ioan Cuza University, 11 Carol I, 700506 Iasi, Romania; cosmin.mocanu@chem.uaic.ro (C.S.M.); gabidr@uaic.ro (G.D.); 3 CERNESIM Centre, Institute of Interdisciplinary Research, Alexandru Ioan Cuza University of Iasi, 11 Carol I, 700506 Iasi, Romania

**Keywords:** NMR spectroscopy, NAP-type peptides, neuroprotection, zinc binding, molecular modeling, Alzheimer’s disease

## Abstract

Aggregation of amyloid-β peptides (Aβ) is a hallmark of Alzheimer’s disease (AD), which is affecting an increasing number of people. Hence, there is an urgent need to develop new pharmaceutical treatments which could be used to prevent the AD symptomatology. Activity-dependent neuroprotective protein (ADNP) was found to be deficient in AD, whereas NAP, an 8-amino-acid peptide (^1^NAPVSIPQ^8^) derived from ADNP, was shown to enhance cognitive function. The higher tendency of zinc ion to induce Aβ aggregation and formation of amorphous aggregates is also well-known in the scientific literature. Although zinc binding to Aβ peptides was extensively investigated, there is a shortage of knowledge regarding the relationship between NAP peptide and zinc ions. Therefore, here, we investigated the binding of zinc ions to the native NAP peptide and its analog obtained by replacing the serine residue in the NAP sequence with tyrosine (^1^NAPVYIPQ^8^) at various molar ratios and pH values by mass spectrometry (MS) and nuclear magnetic resonancespectroscopy (NMR). Matrix-assisted laser desorption/ionization time-of-flight (MALDI ToF) mass spectrometry confirmed the binding of zinc ions to NAP peptides, while the chemical shift of Asp^1^, observed in ^1^H-NMR spectra, provided direct evidence for the coordinating role of zinc in the N-terminal region. In addition, molecular modeling has also contributed largely to our understanding of Zn binding to NAP peptides.

## 1. Introduction

Amyloid-β peptides (Aβ) are protein fragments obtained following the abnormal cleavage assisted by β- and γ-secretase of β-amyloid precursor protein (APP), responsible for the proteinopathy in Alzheimer’s disease (AD) [1,2]. AD is characterized by extensive atrophy of the brain caused by a series of neuropathological changes, such as neuronal loss, the formation of amyloid plaques, development of neurofibrillary tangles, and synaptic loss [3,4]. The misfolding process associated with Aβ aggregation, which is a cause of AD, is largely influenced by metal ions such as Al, Cu, Fe, and Zn that are found in the proximity of senile plaques [5,6].

Zinc is an essential element involved in brain function. This metal can be found mostly tightly bound to zinc-dependent enzymes and other proteins. However, approximately 10% of zinc is not associated with proteins or amino acid ligands. Under pathological conditions, the “free” or “chelatable” zinc accumulates in neurons in neurotoxic amounts. For instance, during intense synaptic activity, zinc accumulates in the synaptic cleft and enters postsynaptic spines, sharing entry routes with calcium [7].

Zinc ions play a critical role in Aβ aggregation according to recent publications and, therefore, become of particular interest in the study of AD [8,9]. Moreover, zinc is the most prevalent transition metal in the brain and performs several functions in physiological processes in both normal and pathological conditions [10,11,12]. Thus, it was observed that high concentrations of zinc coincide with the regions where amyloid plaques form according to the post-mortem investigation of AD patients [13]. Therefore, the induction of Aβ oligomerization is considered as the main contribution of zinc ions to the pathogenesis of AD [14,15]. 

Furthermore, different fragments derived from activity-dependent neuroprotective protein (ADNP) have been studied as potential anti-AD drugs [15,16]. For instance, the neuroprotective ^1^NAPVSIPQ^8^ peptide (NAP) contains two prolines, which confer β conformation. The potential activity of this peptide consist of the inhibition of Aβ(1–40) and Aβ(25–35) fibrillogenesis, protection against Aβ-induced neurotoxicity, and prevention of AD-associated microtubule disruption [17].

The NAP peptide plays an important role in immune regulation, and it was well recognized as a neuroprotective moiety of ADNP [18,19]. Scanning of peptide activity highlighted the neuroprotective role of octapeptide NAP against the toxicity associated with amyloid-β peptide and zinc overload, which causes the breakdown of microtubules in astrocytes and neurons [20,21]. In addition, recent studies showed that the treatment of astrocytes with ZnCl_2_, which results in microtubule disassembly and cell death, was protected by the NAP peptide [20].

While the relationship between heavy metal ions, especially zinc ions, and amyloid-β peptides was recently investigated, little is known about the metal ion binding to neuroprotective molecules, such as NAP-derivate peptides. Therefore, here, we investigated the possible in vitro interaction of zinc ions with two NAP analogs.

Our previous research focused on the design and characterization of NAP-like peptides derived from the native one in order to investigate their behavior in the presence or absence of various metal ions [22,23,24]. Thus, several animal reports investigated the evaluation of neuroprotective strategies. For example, oral administration of Tyr-containing dipeptides, such as Ile-Tyr and Ser-Tyr, in a mouse model influenced catecholamine synthesis and transmission in the brain [25]. Another study suggested that Tyr-Pro dipeptide, capable of crossing the blood–cerebrospinal fluid barrier, can improve impaired cognitive deficits in both working and long-term memories in Aβ-injected AD model mice [26]. In a similar study, a Tyr based Aβ electrochemistry analysis was employed to study the effect of metal ions on Aβ aggregation kinetics. Using the direct oxidation signal of Tyr residue, the authors were able to monitor the metal-induced conformational changes on amyloid-beta peptides in vitro. Thus, the impact of Tyr residue oxidation was correlated to the formation of Zn(II)-Aβ (1–16) complex within a wide pH range from 6 to 9 [27].

The present report focuses mainly on the role of hydroxyl groups, alcoholic and phenolic, in the chelation of Zn^2+^ metal ions. Phenols are known to possess a higher acidic character than alcohols because the negative charge in the phenoxide ion is not localized on the oxygen atom, as it is in an alkoxide ion, but shared by a number of carbon atoms in the benzene ring. Therefore, phenolic hydroxyl of tyrosine is more capable of losing protons and coordinating to the metal ion through oxygen donors than aliphatic hydroxyl of serine [28]. In addition to influencing the binding of metal ions, the presence of an aromatic structure leads to peptide structural rearrangements that may enhance or reduce the peptide neuroprotection activity. Furthermore, according to recent publications, it has been observed that Tyr plays an important role in the complexation and structural modifications of peptides [29]. Oxidative stress is another process involved in the AD pathology [30]. Hence, the Tyr choice was made for this reason as well, since it allowed us to follow the influence on Zn complex formation. In addition, Tyr can be easily oxidized to dihydroxyphenylalanine (DOPA), a precursor used in the treatment of Parkinson’s disease [31]. Thus, following the oxidation process, a new hydroxyl group liable to intervene in metal complexation is introduced. Additionally, when the oxidation process is prolonged, the final reaction product becomes dopaquinone which can easily form metal cluster compounds. Therefore, the complexation process compared to the initial one becomes highly accentuated, with direct consequences in the peptide conformation.

Moreover, it has recently been shown that increasing the hydrophobicity of NAP-derivate peptides enhances the therapeutic effect on microtubule stabilization in AD [32]. Therefore, the addition of a new phenyl group automatically increases the hydrophobicity of the peptide, and therefore, the NAPY (H_2_N-^1^NAPVYIPQ^8^-COOH)-microtubule interaction becomes much more stable due to the hydrophobic system. Additionally, the presence of Tyr residue gives the molecules a much more pronounced rigidity. According to previous studies, replacement of histidine residues from the amyloid beta 1–16 peptide sequence with serine has been shown to increase flexibility. Thus, in aluminum complexation of serine-modified peptides, the aggregation process accelerates with the formation of novel fibrils [33]. Therefore, following this process, the effectiveness of NAP therapeutic treatment decreases drastically.

The molecular mechanisms of neurodegeneration proved to be more complex than a simple association of metal ions with peptides. It also involved the presence of multi-component systems and their time behavior under different pH, ionic strength, and concentration conditions. Consequently, we are searching for a new potential drug for the neurodegenerative disorders due to fibril formation in Aβ plaques in order to address either the prevention or improvement of AD pathophysiology.

In this regard, two synthesized peptides, native NAP fragment and tyrosine-modified peptide NAPY (^1^NAPVYPQ^8^), were incubated at different molar ratios with zinc ions. Their behavior was investigated by NMR spectroscopy and mass spectrometry (MS). Furthermore, the effects of pH on the peptide–metal interaction were also highlighted by NMR spectroscopy, suggesting a higher affinity toward metal ions at basic pH. Moreover, the theoretical calculations were used to confirm the experimental approaches.

## 2. Results

### 2.1. Mass Spectrometric Studies

**Figure 1:**
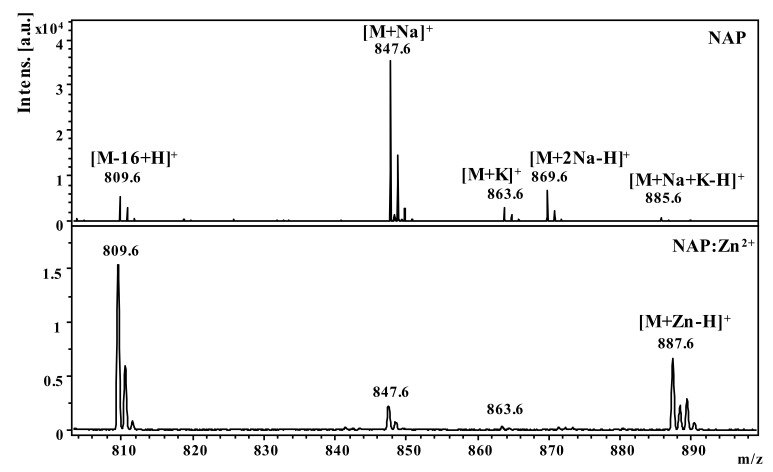
MALDI ToF MS spectra of the NAP peptide in the presence and absence of zinc ions at a 1:10 peptide to metal ratio.

**Figure 2:**
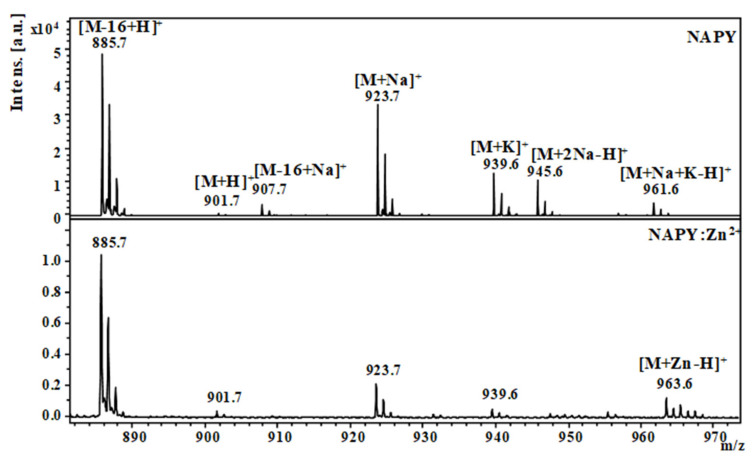
MALDI ToF MS spectra of the tyrosine-modified NAPY peptide in the presence and absence of zinc ions at a 1:10 peptide to metal ratio.

In this study, we examined the interaction of zinc ions (Zn^2+^) with the native NAP peptide and its tyrosine analog NAPY obtained by substituting the fifth amino acid (serine) with tyrosine, in order to investigate the structural changes regarding Zn binding. Thus, the structure of the obtained compound was confirmed by Matrix-assisted laser desorption/ionization time-of-flight (MALDI ToF) mass spectrometry. The detailed characterization of synthesized peptides was previously described in our research [34]. A matrix-assisted laser desorption/ionization time-of-flight mass spectrometer was used to examine the affinity of NAP-like peptides for Zn^2+^ ions (MALDI-ToF MS) [35]. Consequently, Figure 1 and Figure 2 show the NAP-derived peptides positive-ion MALDI-ToF mass spectra before and after adding metal ions (Zn^2+^). The theoretical data from the ChemCalc software was well correlated with the *m*/*z* values found experimentally by the MALDI ToF mass spectrometry (Table 1).

Figure 1 shows the MS spectra obtained for the NAP peptide in the presence and absence of zinc ions. The addition of zinc ions in the sample generated an intense signal at *m*/*z* 887.6 characteristic of Zn^2+^-peptide complexes [M+Zn-H]^+^. The intense molecular peak observed at *m*/*z* 809.6 in both spectra was assigned to the deamidated peptide [M-16+H]^+^ ion. The structure rearrangement that leads to peptide deamidation was favored by the C-terminus glutamine residue. This process takes place during peptide ionization and thus can be considered as a post-source decay mechanism. The most intense signal observed at *m*/*z* 847.6, in the spectrum of native octapeptide generated in the absence of metal ions, was assigned to the sodium adducts ions [M+Na]^+^. Other signals observed in the spectra at *m*/*z* 863.6, *m*/*z* 869.6, and *m*/*z* 885.6 were attributed to the adduct with potassium [M+K]^+^ and two ions of sodium [M+2Na-H]^+^ as well as to [M+K+Na-H]^+^ ions.

In the case of the tyrosine-modified peptide, the MS spectra obtained (Figure 2) showed an intense signal at *m*/*z* 885.7, characteristic of the molecular [M-16+H]^+^ ion. As a result, the presence of Zn^2+^ did not enhance peptide deamination, which was shown in both spectra of peptides with and without metal ions. In addition to the signals corresponding to the deaminated ion, the NAPY spectra clearly showed typical signals of the molecular ion [M+H]^+^ at *m*/*z* 901.7. The signals obtained by the addition of +22 Da and +38 Da were attributed to the adducts of sodium, such as [M+Na]^+^, [M+2Na-H]^+^ and potassium, [M+K]^+^, and [M+K+Na-H]^+^. For the sample incubated with zinc ions, the obtained spectrum showed a distinctive signal at *m*/*z* 963.6, characteristic of the [M+Zn-H]+ ions generated by the Zn^2+^-peptide complex.

### 2.2. NMR Study

The synthesized peptide NAP (H_2_N-^1^NAPVSIPQ^8^-COOH) and NAPY (H_2_N-^1^NAPVYIPQ^8^-COOH) were characterized by NMR spectroscopy in order to perform atom-specific chemical shift assignments and structural information regarding specific metal binding sites. The NMR technique is the method of choice for studying the interaction of metal ions with peptides [36,37,38]. The perturbations generated in the proton NMR spectra, upon Zn^2+^ binding to NAP-like peptides, provide insights about the interaction established between metal ions and various amino acid residues. Furthermore, a comparison between the displayed chemical shifts and the assigned values identified in various peptide studies offers relevant information regarding the ligands involved in coordinating zinc ions.

**Figure 3:**
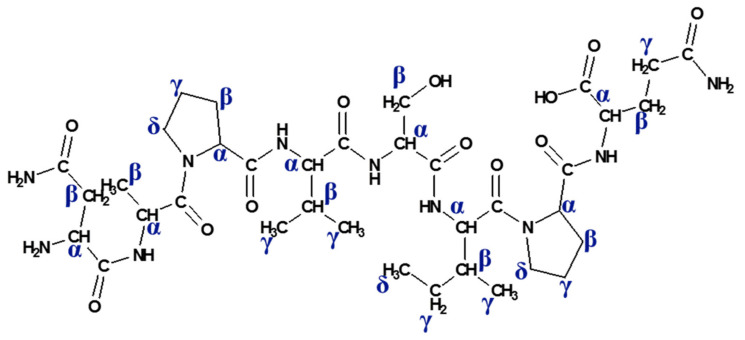
Two-dimensional structure of native NAP peptide (Asn-Ala-Pro-Val-Ser-Ile-Pro-Gln) with the marked alpha (α), beta (β), gamma (γ), and delta (δ) protons.

In this regard, one-dimensional proton spectra of the native NAP peptide and tyrosine-modified NAPY peptide dissolved in deuterium oxide were recorded. All the signals measured were assigned to the corresponding protons according to the observed chemical shift, homonuclear correlation (^1^H–^1^H correlation), and heteronuclear direct correlation (^1^H–^13^C correlation). Additionally, the ^13^C spectrum presented a suitable signal dispersion, which allowed the assignment of carbon atoms that are directly bound to the protons. Although some signals were affected by an exchange process with the deuterium atoms of the solvent and are missing in the spectra, such as amide, amines, hydroxyl, and carboxylic protons, the NMR experiments confirmed the amino acid sequence. The observed protons are defined in the following structure (Figure 3).

**Figure 4:**
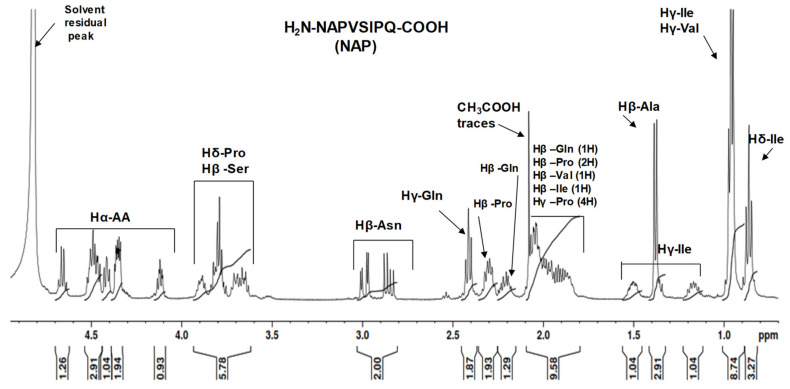
^1^H-NMR spectrum of NAP peptide dissolved in D_2_O solvent.

The ^1^H-NMR spectrum of NAP peptide (Figure 4) showed signals in the aliphatic region between 0.2 and 4.7 ppm. The most deshielded signals belonged to the eight alpha protons Hα defined in the peptide structure (amidic protons are not visible in the spectrum). The characteristic signals of Hβ protons of serine (2H) and Hδ pyrrolidine protons of proline (4H) were observed in the range of 3.6–3.9 ppm. Due to the spatial arrangement of the atoms in the molecule, the Hβ protons of Asn generated two signals at 2.98 ppm (1H) and 2.85 ppm (1H). Other Hβ protons corresponding to the Val (1H) and Ile (1H) along with Hγ protons (4H) from the pyrrolidine ring generated signals between 2.1 ppm and 1.8 ppm. In the case of glutamine, the Hβ protons were observed at 2.20 and 1.98 ppm (2H), while Hγ protons generated signals identified at 2.41 ppm (2H). The intense signal observed as a doublet at 1.36 ppm was easily attributed to the methyl protons (3H) of alanine, and the signals found at 1.47 ppm and 1.15 ppm corresponded to the Hγ isoleucine protons (2H). Additionally, the most shielded protons were the methyl group of valine (Hγ) and isoleucine (Hγ, Hδ) that generated signals in the range of 1.5–0.8 ppm. Furthermore, the intense signal observed at 2.06 ppm was due to acetic acid traces from the sample reagent used in the peptide elution step.

**Figure 5:**
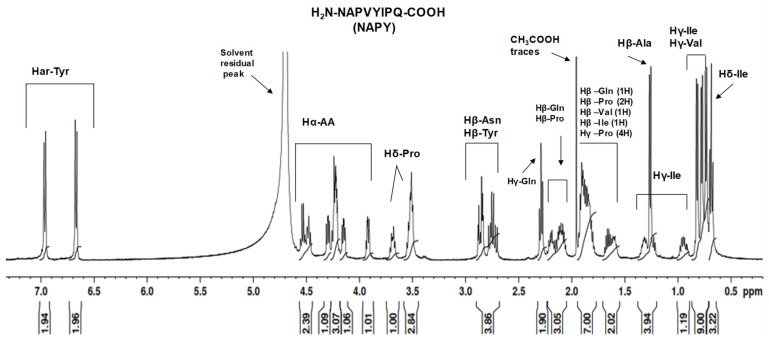
^1^H-NMR spectrum of NAPY peptide dissolved in D_2_O solvent.

In the case of NAPY, the proton NMR spectrum (Figure 5) contains structural similarities compared to the previously investigated one (Figure 4). However, small differences caused by the replacement of the fifth amino acid were observed in the ^1^H-NMR spectra. For example, signals corresponding to the protons attached to the phenolic nucleus of tyrosine were observed in the aromatic region at 7.07 ppm (2H) and 6.78 ppm (2H). Additionally, the signal given by the tyrosine alpha proton was identified at 4.59 ppm (1H), while the Hβ proton (2H) overlapped the signals generated by the beta protons of asparagine in the region of 3.03–2.77 ppm. Another notable change was the rearrangement of signals generated by gamma protons found in the structure of the Ile and Val amino acids. Thus, the observed aspects highlight the structural changes suffered by the peptide following the replacement of serine with tyrosine and the influence that the new amino acid has on the peptide structural rearrangement.

After characterization of pure peptides, the metal ion binding capacity to NAP octapeptides was investigated by NMR titration. Thus, solutions of peptide and Zn(II) ions at a molar ratio equal to 1:0.1, 1:0.5, 1:1, 1:1.5, 1:2, and 1:10 were prepared by gradually adding 5 µL of metal ion stock solutions (60, 480, 2400, or 9600 mM in H_2_O) to the peptide solution (10 mM in D_2_O). The mixture series of metal ions and peptides were transferred into standard 5 mm NMR tubes and all experiments recorded at 25 °C.

**Figure 6:**
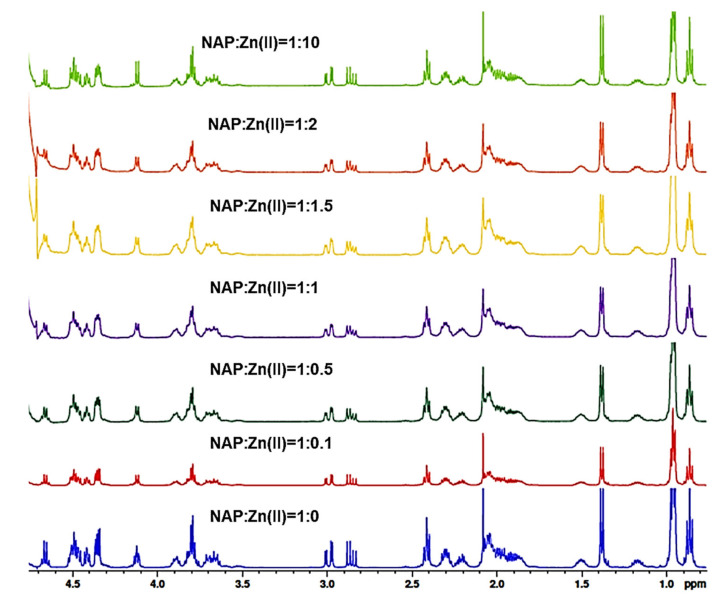
^1^H-NMR spectrum in the aliphatic region of NAP peptide titrated with increasing amounts of Zn^2+^ at pH = 5.

**Figure 7:**
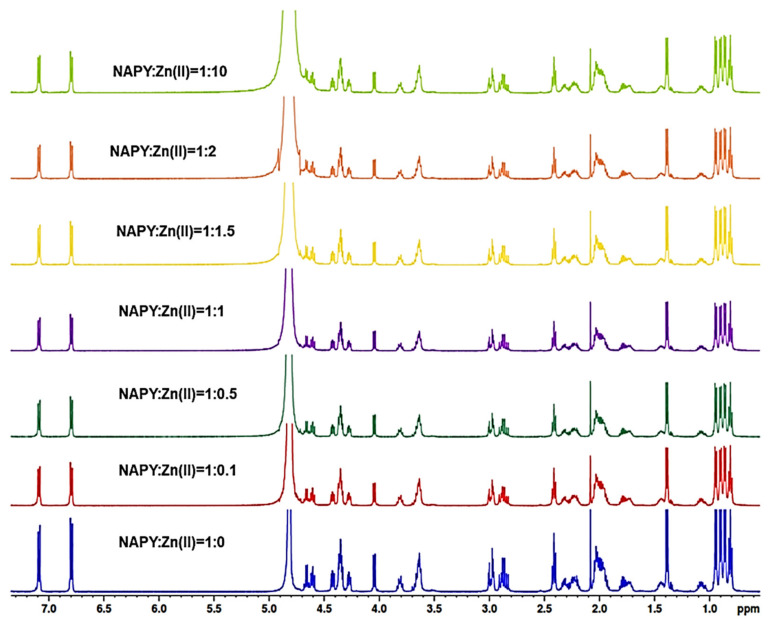
^1^H-NMR spectrum in the aliphatic region of NAPY peptide titrated with increasing amounts of Zn^2+^ at pH = 5.

The figures which show the 1D proton NMR spectrum of NAP (Figure 6) and NAPY (Figure 7) peptides titrated with increasing amounts of Zn^2+^ at pH 5 are presented below. The addition of Zn^2+^ ions did not cause a perturbation of ^1^H-NMR resonances at the natural pH of the solution upon adding ZnSO_4_ to peptides. Thus, no chemical shifts were observed upon metal increasing, suggesting the presence of only intermediate exchange regime. 

A similar pattern was observed in Figure 7. According to these results, the ^1^H-NMR spectrum incorporating Zn^2+^ titrations of tyrosine modified NAPY peptide did not cause any perturbation to the system.

**Figure 8:**
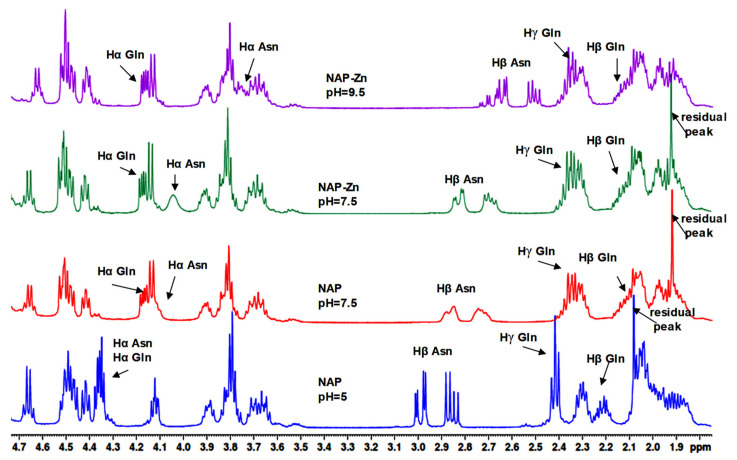
^1^H-NMR spectra of NAP peptide titrated with Zn^2+^ ions at pH 7.5 and pH 9.5, at a 1:10 molar ratio.

**Figure 9:**
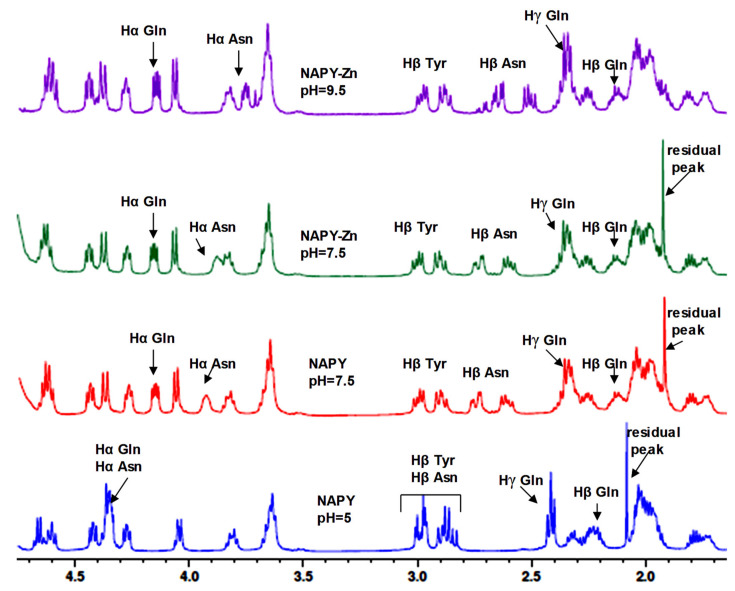
^1^H-NMR spectra of NAPY peptide titrated with of Zn^2+^ ions at pH 7.5 and pH 9.5, at a 1:10 molar ratio.

On the other hand, increasing the pH value of solution caused perturbation of NMR resonances (Figure 8 and Figure 9). Thus, at pH 9.5 and a 1:10 molar ratio of peptide:Zn^2+^ titration, new exchange cross peaks and shifts appeared in ^1^H- NMR spectra. The pH was adjusted by adding crystals of potassium carbonate K_2_CO_3_ and was measured before recording each spectrum. Both peptides presented a significant chemical shift deviation for the side-chain peak of asparagine residues (Hβ protons). Thus, upon addition of zinc ions, Hα protons from Asn and Gln residues generated a noticeable chemical shift.

All changes caused by affected protons appear as upfield signals in the spectrum. Additionally, both peptides provided a direct indication regarding the involvement of the N-terminus and C-terminus in zinc binding. Therefore, the nitrogen atoms of the amino group oxygen from the carbonyl group can be envisaged as possible coordination sites for metal ions. The chemical shift changes are presented in Table 2 for NAP and Table 3 for the NAPY-modified peptide.

In the case of the NAP peptide, in addition to the mentioned modifications, the Hα protons from alanine generated chemical shift changes at pH 9.5. Thus, considering the position of Ala in the peptide structure (near the N-terminal), it can be concluded that the changes are not purely caused by conformational alterations, but by a direct (de)shielding effect of the positively charged zinc ion found nearby. Moreover, there were no changes in the chemical displacement of protons in the range of 0.8–1.6 ppm (Hβ in Ala, Hγ in Ile and Val, Hδ in Ile) and 3.6–4.7 ppm.

An evident change of the peptides NAP and NAPY conformation in solution was visibly recorded in the ^1^H-NMR spectra at pH 7.5. Chemical shifts appear in protons from asparagine and glutamine residues. In experiments conducted with zinc ions and peptides at pH 7.5, new modifications were observed in the alpha protons from the asparagine amino acid.

At pH 9.5, the tyrosine beta resonances from the NAPY peptide were unchanged upon zinc addition, while the aromatic protons displayed light chemical shift changes (Figure 9) upon shielding effect. Thus, Zn^2+^ slightly affects the aromatic protons from Tyr5, appearing to lower the chemical shift. These observations highlight the important role played by Y5 hydroxyl in binding zinc ions. Other chemical shift changes observed in the ^1^H-NMR spectra of the NAPY peptide were similar to that suffered by the NAP peptide.

### 2.3. Molecular Modeling

Oftentimes, the atomic-level details of the structures of pharmaceutically relevant receptors are not available. Three-dimensional alignment of potential ligands can be utilized to derive structural requirements for biological activity in such instances. Another strategy is pharmacophore elucidation, in which several ligands are aligned, and a small collection of essential molecular features required for biological activity is derived from the alignment [39]. Additionally, another method is to search a database for ligands with one or more conformations which superpose well with a query molecule using 3D molecular alignment [40]. Methodologies based on 3D alignment for discovering biologically active ligands employ the qualitative premise that if two ligands have similar biological activity and bind in similar modes, then their bound conformations match well, and inferences about the nature of the receptor may be drawn.

**Figure 10:**
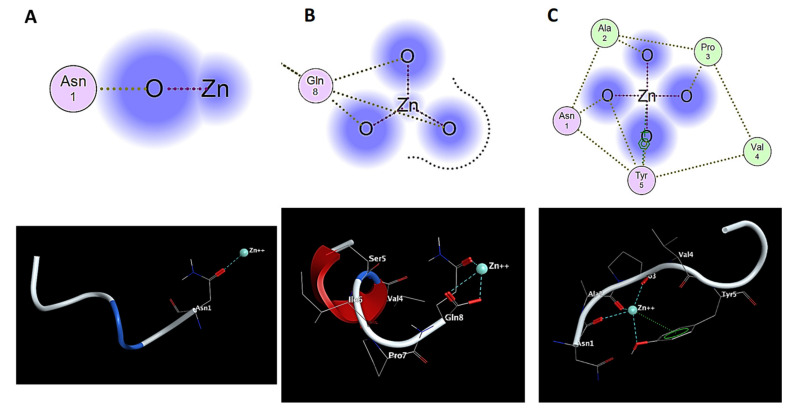
Flexible alignment of Zn^2+^ interaction, with NAP (**A**,**B**) and NAPY (**C**), performed with Molecular Operating Environment software.

In order to develop and confirm the interaction of zinc ions with the sequence of the NAP and NAPY peptide, we resorted to a series of theoretical approaches. First, we applied Flexible Alignment using Molecular Operating Environment 2016.02 (MOE) in order to find the coordination pattern of Zn^2+^ for the NAP and NAPY peptide. According to the submitted simulation, the zinc ion binds to the NAP through Asn^1^ or Gln^8^ at the C-terminal or N-terminal of the peptide (Figure 10A,B). These bindings were also noticed in the NMR spectra, confirming the experimental procedure. However, these representations suggest that the interaction between Zn^2+^ and the NAP sequence is not well defined, as in the case of the NAPY-Zn^2+^ complex (Figure 10C).

Furthermore, according to Figure 10C, the zinc ions bind strongly with the NAPY peptide structure through a cluster formed by Asn^1^, Ala^2^, Pro^3^, Val^4^, and Tyr^5^. Moreover, besides the oxygen atom, Tyr^5^ seems to contribute even with its backbone through the π-cation interaction, acting as a stabilizer to the Zn^2+^-NAPY complex. Hence, in this case, the binding potential between the zinc ion and the tyrosine analogue becomes stronger. 

In addition, the NAP/NAPY-Zn^2+^ complex was subjected to molecular dynamics (MD) in order to investigate the stability and the binding interaction between zinc ions and the corresponding amino acid under thermic conditions with the same software. The purpose of this approach was to study the influence of body temperature (37 °C) regarding the distances between Zn^2+^ and the atom involved in the coordination process. Hence, a series of parameters described in the Section 4.2.3 was applied using three different methods regarding MD algorithms: NPA (Nosé-Poincaré-Andersen), NHA (Nosé-Hoover-Andersen), and BER (Berendsen equations) (Figure S1, Supplementary Materials).

The coordination binding, as well as the structure of the peptide under 25 °C (298.15 K) for complex stabilization and 37 °C (310.15 K) for the body temperature effect, is represented in Figure S2 in the case of NAP-Zn^2+^-Asn^1^, in Figure S3 for NAP-Zn^2+^-Gln^8^, and in Figure S4 for the case of NAPY and zinc ion binding (all in Supplementary Materials).

According to the MD runnings, there are few modifications regarding the binding pattern of NAP and NAPY peptides under a thermic condition at 25 degrees (room temperature) and 37 degrees (body temperature). In the case of the NAPY peptide, under a thermic condition, it was noticed that the Tyr plays an important role in metal complexation. According to Figure S3, the oxygen atom of Tyr influences the stability of Zn^2+^ complexation. At 37 degrees, it seems that the coordination binding is stronger than at 25 °C. This suggests that in the human body, the NAPY-Zn^2+^ complex becomes more stable. Moreover, these data also suggest that the peptide has a strong stability which did not influence the binding cluster with zinc ions.

Consequently, significant changes were also analyzed regarding binding sites and the distance between zinc ions and the corresponding atom from the amino acid involved in the binding process (Table S1, Supplementary Materials).

According to these data, relevant modifications were observed regarding the distance between zinc ions and the amino acid from the NAP/NAPY sequence. As can be noticed, under a thermic condition compared with the static momentum, the bond between Zn^2+^ ions and the atom involved in the coordination process becomes stronger at 37 degrees (310.15 K). This suggests that the temperature plays an important role in the metal ion binding in our case. Moreover, it also can be observed that in the coordination process, the oxygen in the sp^2^ state strengthens the bond more than in the sp^3^ state. Therefore, according to these data, the more oxygen the peptide has in the sp^2^ state, the stronger the bond between the metal and the peptide becomes regarding the temperature.

In addition, it was noticed that in the case of NAP-Zn^2+^-Asn^1^, there was a slight dissociation of the zinc ion when it was applied to a higher temperature. Furthermore, in the NAP-Zn^2+^Gln^8^ complex, there were no major differences in the distance between the zinc ion and the corresponding atom when the temperature increased from 25 to 37 degrees—just a minor drop of binding energy. This suggests that in this situation, at body temperature, the bonds in the coordination process become stronger, even if the distances remain almost the same. In the case of tyrosine-modified peptides, the bond between O^sp2^(Asn^1^) and zinc increases while the distance that implies alanine and proline becomes shorter, and the bond energies are constant.

Thus, according to these data, at 37 degrees (310.15 K), there is a higher probability to form a NAP-Zn^2+^Gln^8^ complex than NAP-Zn^2+^-Asn^1^. Moreover, the addition of tyrosine increases the stability of the zinc ions complex though alanine and proline residues, as well as the oxygen and the π-cations of this amino acid (stability is confirmed in Figure 10 and Figure S4).

Hence, this approach could have an important implication in drug design with direct consequences in peptide stability and blood–brain barrier permeability. Furthermore, these observations were well correlated with NMR investigations, suggesting that the data obtained in our study were accurate according to the experimental and theoretical approaches. Therefore, our experimental and theoretical design helped us to reveal the underlying structural modification under thermic and metal conditions.

## 3. Discussion

The antioxidant octapeptide NAP acts as a therapeutic agent at femtomolar concentrations, enhancing neuronal development, learning, and memory [41]. This peptide has been applied in different clinical studies, showing cognitive and functional protection in research trials involving patients with AD-specific cognitive impairment [42]. Moreover, it has been shown to inhibit Aβ aggregation, while the neuroprotective mechanism exerted by NAP assumes the folding modulation of toxic proteins in the extracellular environment [18].

The etiology of this pathology is related to the formation of fibrils and protein aggregates associated with metal binding, which leads to conformational changes in the morphology of the entire brain [43].

Although the homeostasis of metal ions, such as iron, copper, zinc, and calcium, in the brain is crucial for maintaining normal physiological functions, the metabolism alteration of these ions in the brain is closely linked to the onset and progression of AD [44].

Various studies showed that zinc is involved in several AD-associated processes, such as neurotransmission, nonamyloidogenic APP processing, enzymatic degradation of the Aβ peptide, the Aβ–DNA interaction, and inducing Aβ oligomerization [12,14]. Furthermore, the zinc ion concentration in amyloid plaques was demonstrated to be high, reaching 1 mM [45]. Thus, chelating agents capable of linking together metal ions to form complex ring-like structures are of particular interest for reducing metal toxicity.

The zinc binding site to the amyloid peptide is located at the disordered N-termini reach in aspartate (Asp1, Asp7), glutamate (Glu3, Glu11), and histidine residues (His6, His13, and His14) [46]. NMR studies performed in aqueous solution at pH 6.5 and 7.4 using the 1–16 amyloid fragment showed that Zn^2+^ was bound to the three histidine residues and to the two carboxylic groups of Glu11 [47]. Another NMR study, performed on Aβ 1–28, reveled a penta-coordination of Zn^2+^ by His6, Glu11, His13, His14, and Asp1 at the N-terminus [48].

At low pH, where the majority of the amino groups are protonated, the affinity towards the metal ions was weak, and the stability of the complex was plain. As the pH rises, the affinity and stability of the peptide–metal complexes increase. This suggests that at low pH, almost no metal ions are complexed. However, once the pH increases, the peptide–metal affinity reaches a maximal value. This process indicates the possibility of a recovery process by readjusting the pH [49].

There are five potentially active amino acid residues in human Aβ peptides: three His at positions 6, 13, and 14; one Met at position 35; and one Tyr at position 10 [50]. The results obtained after increasing the pH value showed a higher affinity toward metal ions. Moreover, similar studies on zinc chelation agents showed that water-soluble macroligands had the best affinity at basic pH (pH 9), while acidic pH promotes competition between H^+^ and divalent metal ions [49].

In addition, the structural changes of the Zn-NAP/NAPY complex could also be attributed to the solvation of the system. Ben-Naim highlights this process by establishing that the effect of a pair of functional groups (hydroxyl or amino), either intermolecular or intramolecular hydrogen bonds, depends on the total standard free energy of the peptide folding [51]. Moreover, a similar study reached the same conclusions. The relevance of the solute–solvent coupling, as well as the differences in behavior of hydrophilic and hydrophobic moieties, depends on the amino acid residues [52].

Additionally, these interferences could become significant in our case because they might interfere with the aggregation process in AD. A model has been used to investigate peptide aggregation at hydrophobic–hydrophilic interfaces and compare it to aggregation in a clear water solvent [53]. It was also relevant in a study which investigated the size and fractal character of the monomers, oligomers, and fibrils of the various amyloid-beta peptides using the small angle neutron scattering method (SANS) [54]. Using molecular dynamics simulation, new insight regarding the influence of water molecules on the folding landscape of amyloid peptides was obtained. Thus, in the presence of water, hydrophobic regions were shown to accelerate fibril construction while hydrophilic sequences reduced the growth of fibrils by water-stabilizing metastable intermediates [55]. The relation between hydrogen bonding and the hydrophobic sequence leading to protein folding is predominantly determined by the backbone intermolecular hydrogen bonding interactions [56]. Hydrogen bonds have significant roles in protein assemblies and in the organization of biomolecular protein–ligand complexes. All amino acids can generate hydrogen bonds and serve as both donors and acceptors. Additionally, the hydroxyl group of tyrosine can undergo hydrogen bonding with the backbone amide of nearby tyrosine residue, leading to structure stabilization [57]. Hydrogen bonding events can vary at different pH conditions. For example, collagen-like peptides exhibit a reduced intramolecular hydrogen bonding behavior at basic condition and a minimal event of H-bond between amino acid residues and water molecules at acid conditions [58]. Moreover, it was proved that in different protein aggregations, water performs an important role [55]. Therefore, the Zn-NAP/NAPY complexations and the obtained results could be attributed to the solvent, due to the hydrophobic and structure stabilization.

## 4. Materials and Methods

### 4.1. Peptide Synthesis

The octapeptides NAP (NH_2_-^1^NAPVSIPQ^8^-COOH) and NAPY (NH_2_-^1^NAPVYIPQ^8^-COOH) were synthesized using the solid phase Fmoc/tBu solid phase synthesis (SPPS). The peptide synthesis was carried out manually in a linear fashion on a Fmoc-Gln(Trt)-Wang Resin and was described in our previous work [28]. Summarily, the manual peptides synthesis was performed in a fritted plastic reactor attached to a vacuum pump in order to remove the washing solutions. This synthesis was performed in dimethylformamide (DMF) solvent. Protecting groups such as Fmoc or tert-butyl, used to protect the amino acids at the N-terminus or the side chain, were removed with 20% piperidine in DMF. Final cleavage of the peptides from the resin was performed with a TFA: TIS: H_2_O solution, in a ratio of 95:2.5:2.5 (*v*/*v*/*v*). The precipitation of peptides was performed using diethyl ether (−20 °C). The resulting fractions, eluted with 5% acetic acid solution in ultrapure water, were lyophilized. The ZnSO_4_·7H_2_0 salt used as metal ion source was procured from Sigma Aldrich (Germany). All solutions were prepared using deionized water (18.2 MΩ·cm) from a Milli-Q system (Millipore, Bedford, MA, USA).

### 4.2. Instruments

#### 4.2.1. Mass Spectrometry

The matrix-assisted laser desorption ionization time-of-flight (MALDI-ToF) MS analysis was performed on a Bruker Ultraflex MALDI ToF/ToF mass spectrometer operated in positive reflectron mode and equipped with a pulsed nitrogen UV laser (λmax 337 nm). For MS studies, metal complexes of NAP-like peptides obtained by mixing the corresponding octapeptides with metal solution at physiological pH (pH 7.4) at a 1:10 peptide-to-metal ratio were used. MilliQ-grade water was used to make the stock solutions of peptides (8 mM) and metal ions (80 mM). The samples were loaded onto a 384-spot target plate of the MALDI-ToF device using the dried-droplet method. On the target, the sample and matrix solution (α-cyano-4-hydroxycinnamic acid dissolved in a solution comprising 2:1 ACN: 0.1% TFA in MilliQ) were mixed and allowed to dry. After co-crystallization, the metal plate was introduced into the mass spectrometer and subjected to short laser pulses. The acquired signals were expressed as atomic mass units (Da). Finally, the obtained spectra were processed using Bruker’s FlexAnalysis 3.4 software.

#### 4.2.2. NMR Spectroscopy

Proton NMR experiments were recorded using a Bruker Avance III, 500 MHz frequency spectrometer, equipped with a 5 mm Pabbo detection probe and operating at 500 MHz for ^1^H nucleus and 125 MHz for ^13^C nucleus. Chemical shifts are reported in δ units (ppm), relative to the internal standard (TSP, δ 0.0 ppm). The experiments were recorded without introduction of a pre-saturation pulse for solvent signal suppression. All spectra were recorded at 298 K with 12 mg/mL peptide dissolved in D_2_O with 5 mM TSP 3-(trimethylsilyl)propionic-2,2,3,3-d_4_ acid, sodium salt in D_2_O added, as the internal chemical shift calibration standard. Due to the dissolution of the peptide in deuterium oxide, in the ^1^H–NMR spectra, some signals are affected by a process of exchange with deuterium in the solvent and are missing in the spectra; for example, the exchange of amidic protons, amino protons from asparagine and glutamine residues, and hydroxyl hydrogens from serine and tyrosine residues. Based on the peak integral measured in the quantitative 1D ^1^H-NMR spectrum, the number of protons in the peptide was verified. NMR data were processed and analyzed using BrukerTopSpin3.2. software.

#### 4.2.3. Data Analysis

In this study, we investigated the binding pattern changes of NAP and NAPY peptides under the influence of Zn^2+^ ions using different theoretical approaches. For this purpose, the Molecular Operating Environment 2016.02 modeling software from Chemical Computing Group (MOE) was used [59]. First, the peptides (^1^NAPVSIPQ^8^—NAP and ^1^NAPVYIPQ^8^—NAPY) were built and subjected to energy minimization in order to calculate atomic coordinates which are local minima of a potential energy function. The Amber10:EHT forcefield was used with van der Waals and electrostatic forces considered. Furthermore, tethers, distances, angles, and dihedrals were restrained. All atoms were calculated with partial charges and cutoff of (8,10). The Hamiltonian was PM3-RHF with al spin state. The gradient was set at 0.05 RMS kcal/mol/A^2^, and regarding quantum mechanics, the basis set was AM1, and the multiplicity singlet. These restraints were removed upon termination of the energy minimization [60,61,62].

Subsequently, the NAP/NAPY-Zn^2+^ system was subjected to Flexible Alignment, used in general for flexibly aligning small molecules. This algorithm takes a collection of small molecules with 3D coordinates as input and generates a set of alignments. Our purpose was to set a coordination binding to the assigned peptide. Each alignment obtained a score that measured the quality of the alignment in terms of both overlap and internal strain of molecular features. The probability density functions used in our case were Gaussians. The iteration limit was set at 1000 and a failure limit at 50. The energy cutoff was set at 15. Moreover, forcefield charges were calculated prior to search and the stochastic conformations [63,64,65].

The ligand interactions were used to diagrammatically depict the active site of the NAP/NAPY-Zn^2+^ complex. These interactions were obtained according to the method described in the literature [66]. In our case, the hydrogen bonds, metal ligation, and nonbonded residues were taken into account. Regarding this protocol, a series of different steps were followed: the chosen ligand was set as the central focus of the diagram, the ligand’s coordinates were determined, the residues were placed to form strong hydrogen bonds with the ligand, the residues that are close to the ligand with weaker interactions were placed, and proximity contour and solvent accessibility were calculated [66,67,68].

Consequently, the NAP/NAPY-Zn^2+^ system was further subjected to molecular dynamics (MD) in order to analyze the influence of thermic condition in metal–peptide complexation. Three independent MD runnings were provided using NPA (Nosé-Poincaré-Andersen), NHA (Nosé-Hoover-Andersen), and BER (Berendsen equations) algorithms. For each MD procedure, a run time of 1000 ps (picoseconds, or 1 ns) was set followed by an equilibrium stage of 100 ps at 298.15 K and 310.15 K—a total run time of 2.2 ns for each algorithm. The same forcefield was used as in the minimization of the system energy, Amber10:EHT with a cutoff of (8,10). The constrains were set at light bonds, and tethers, distances, angles, and dihedrals were restrained. The checkpoint of the MD run was set at 250 ps, with a time step of 0.002 ps [69,70,71].

## 5. Conclusions

In recent years, molecular studies have become a useful tool in medicinal chemistry, furnishing relevant information concerning binding sites and binding energy. According to our study, the data obtained showed that zinc ions bind not only to amyloid-β peptides but also to the neuroprotective molecules, such as the native NAP peptide and its analog obtained by replacing the serine residue from the NAP sequence with tyrosine (^1^NAPVYIPQ^8^). Furthermore, it was observed that at different molar ratios and pH values, the interaction between Zn and our investigated peptides changing and the N-terminal sequence of peptides was involved in ion binding. Moreover, the inquiry of mass spectrometry and NMR spectroscopy confirmed the binding of the zinc ion to NAP peptides, while the chemical shift of Asp1, observed in ^1^H-NMR spectra, provided direct evidence for the coordinating role of zinc in the N-terminal region. In addition, the molecular modeling showed a perspective regarding their interactions, confirming the experimental data obtained during mass spectrometry and NMR investigations.

Hence, our study offers a unique insight into metal ion interactions with the NAP and NAPY peptide by analyzing the influence of Tyr swapping with Ser, more specifically the influence of the benzene nucleus. Our approaches demonstrated that the addition of a phenyl group increases the binding potential of Zn ions to the structure of peptides. Therefore, the investigation of such short NAP peptide fragments and analogues provides improvements in the structure and biocompatibility of peptide-based drugs enhancing the therapeutic pathways of neuropathologies and can also help to assess chemical phenomena in peptide–metal ion complexes to understand the modifications and structural effects of peptides with therapeutic activity. All these bring important information for understanding AD. Thus, further studies aiming to obtain new modified peptides starting from the active site of the NAP peptide, which is represented by the SIP center, are needed in order to better understand the reactivity of the interactions between metal ions and peptides.

## Figures and Tables

**Table 1:** The primary structure and molecular weight of the NAP/NAPY peptides in the presence of zinc ions (Zn^2+^), as experimentally determined with a MALDI-ToF instrument or calculated with ChemCalc online software.

Peptide	Molecular Ion	Theoretical (*m*/*z*)	Experimental (*m*/*z*)
NAP (H_2_N–NAPVSIPQ-COOH)	[M-16+H]^+^	809.4	809.6
	[M+H]^+^	825.4	825.6
	[M+Na]^+^	847.4	847.6
	[M+K]^+^	863.4	863.6
	[M+2Na-H]^+^	869.4	869.6
	[M+Na+K-H]^+^	885.4	885.6
	[M+Zn-H]^+^	887.4	887.6
NAPY (H_2_N–NAPVYIPQ-COOH)	[M-16+H]^+^	885.5	885.7
	[M+H]^+^	901.5	901.7
	[M+Na]^+^	923.5	923.7
	[M+K]^+^	939.4	939.6
	[M+2Na-H]^+^	945.4	945.6
	[M+Na+K-H]^+^	961.4	961.6
	[M+Zn-H]^+^	963.4	963.6

**Table 2:** Assignment of signal modifications in the ^1^H-NMR spectra of NAP peptide at different values of pH and in the presence of zinc ions.

Chemical Shift (ppm)	NAP pH 5	NAP pH 7.5	NAP-Zn pH 7.5	NAP-Zn pH 9.5
^1^Hα Asn	4.34	4.10	4.04	3.77
^1^Hβ Asn	2.99	2.87	2.82	2.65
	2.85	2.72	2.69	2.51
^1^Hα Gln	4.36	4.17	4.17	4.17
^1^Hβ Gln	2.20	2.13	2.13	2.13
^1^Hγ Gln	2.41	2.36	2.36	2.36

**Table 3:** Assignment of signal modifications from ^1^H-NMR spectrum of NAP peptide at different value of pH and in the presence of zinc ions.

Chemical Shift (ppm)	NAPY pH 5	NAPY pH 7.5	NAPY-Zn pH 7.5	NAPY-Zn pH 9.5
^1^Hα Asn	4.33	3.39	3.87	3.75
^1^Hβ Asn	2.99	2.74	2.73	2.65
	2.85	2.64	2.59	2.51
^1^Hα Gln	4.34	4.15	4.15	4.15
^1^Hβ Gln	2.22	2.13	2.13	2.13
^1^Hγ Gln	2.41	2.34	2.34	2.34

## Data Availability

The data presented in this study are available in article and Supplementary Materials.

## Supplementary Materials

The following are available online at https://www.mdpi.com/article/10.3390/ph14101011/s1. Figure S1 —The structures at 310.15 K following molecular dynamics using 3 algorithms of NAP/NAPY-Zn^2+^ complexes; Figure S2—Molecular dynamics of NAP-Zn^2+^-Asn^1^ complex; Figure S3—Molecular dynamics of NAP-Zn^2+^-Gln^8^ complex; Figure S4—Molecular dynamics of NAPY-Zn^2+^; Table S1—The molecular parameters of NAP/NAPY-Zn2+ complex according to the MD simulation.
