# Supplementary material for: Zinc Binding to NAP-Type Neuroprotective Peptides: Nuclear Magnetic Resonance Studies and Molecular Modeling
Source: Pharmaceuticals (Basel). 2021 Oct 1;14(10):1011. doi: 10.3390/ph14101011 (PMC8541368; doi:10.3390/ph14101011)
Supplement: Supplementary file 1 [file pharmaceuticals-14-01011-s001.zip › pharmaceuticals-1344314-supplementary.pdf]

Supporting Information

Article

Zinc Binding to NAP-Type Neuroprotective Peptides: Nuclear Magnetic Resonance Studies and Molecular Modeling

Ancuta-Veronica Lupaescu[1], Cosmin Stefan Mocanu[2], Gabi Drochioiu[2] and Catalina-Ionica Ciobanu[3,*]

[1] MANSiD Research Center, Stefan cel Mare University, 13 University, Suceava-720229, Romania;
ancuta.lupaescu@usm.ro (AVL)
[2] Faculty of Chemistry, Alexandru Ioan Cuza University, 11 Carol I, Iasi-700506, Romania; cosmin.mocanu@chem.uaic.ro; (CSM) gabidr@uaic.ro (GD)
[3] Institute of Interdisciplinary Research-CERNESIM Centre, Alexandru Ioan Cuza University of Iasi, 11 Carol I, Iasi, 700506, Romania; catalina.ciobanu@uaic.ro (CIC)
Corresponding authors: catalina.ciobanu@uaic.ro (CIC)

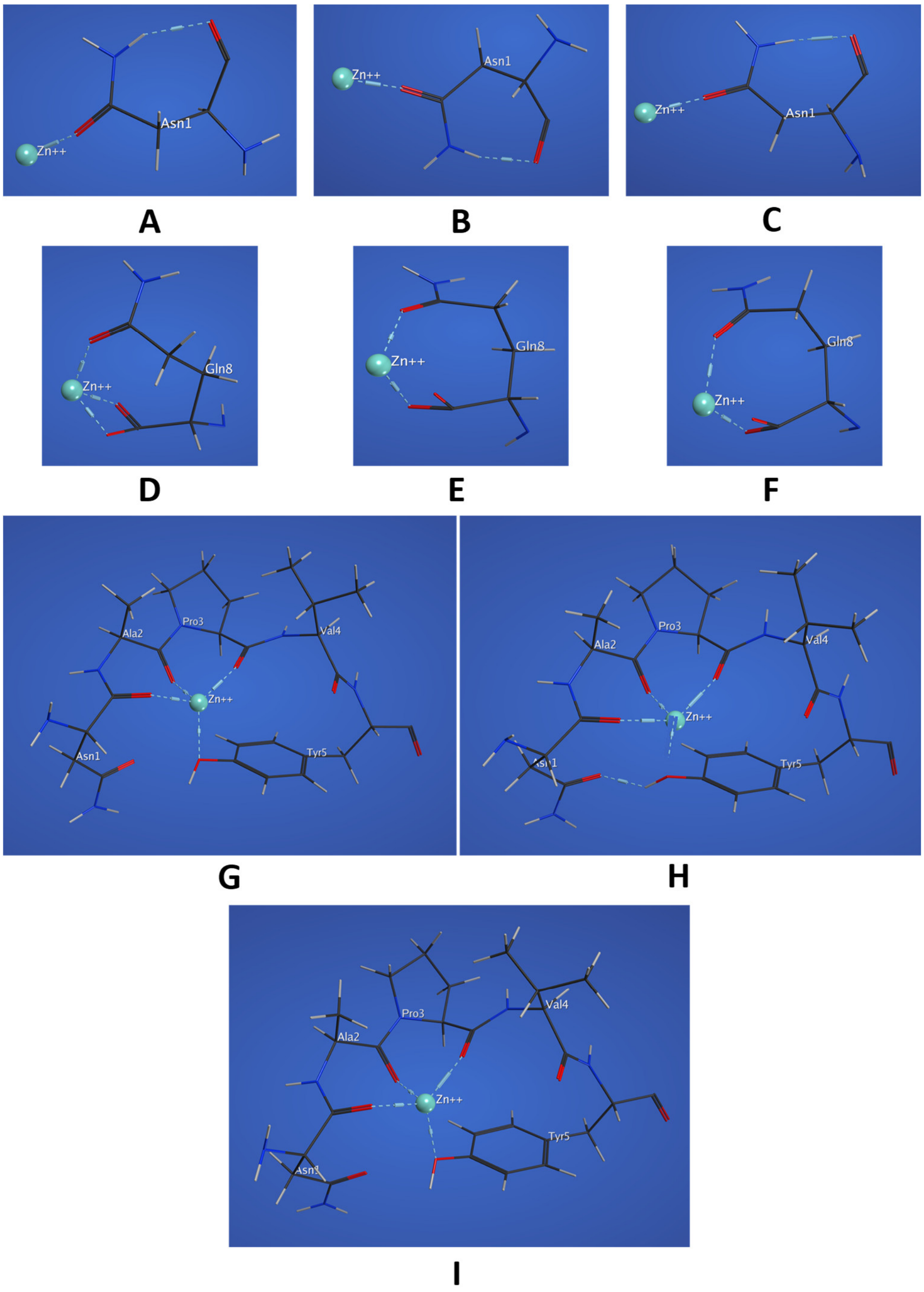

Figure S1. The structures at 310.15 K following molecular dynamics using 3 algorithms: (A) NAP-Zn^{2+}-Asn^{1} with NPA, (B) NAP-Zn^{2+}-Asn^{1} with NHA, (C) NAP-Zn^{2+}-Asn^{1} with BER, (D) NAP-Zn^{2+}-Gln^{8} with NPA, (E) NAP-Zn^{2+}-Gln^{8} with NHA, (F) NAP-Zn^{2+}-Gln^{8} with BER, (G) NAPY-Zn^{2+} with NPA, (H) NAPY-Zn^{2+} with NHA and (G) NAPY-Zn^{2+} with BER.

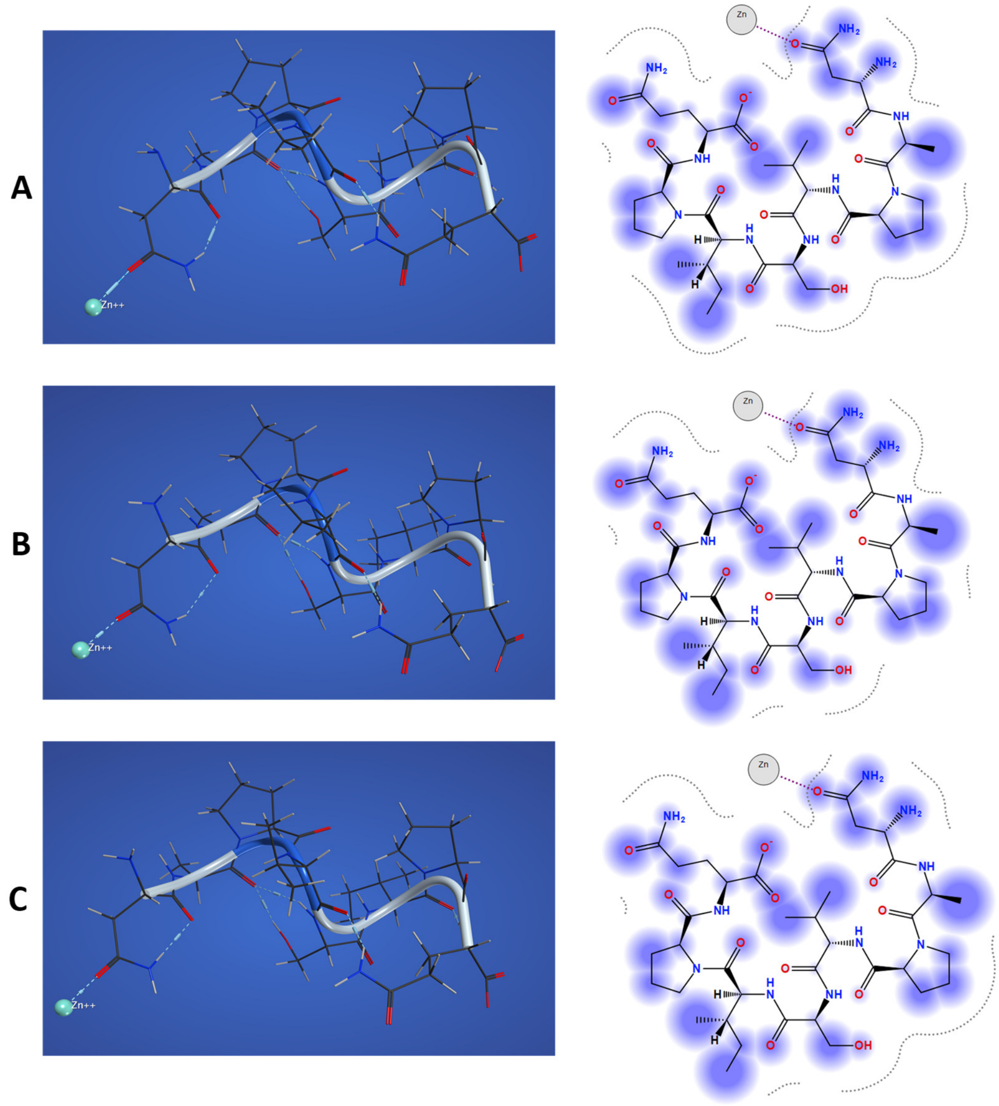

Figure S2. Molecular dynamics of NAP-Zn^{2+}-Asn^1 complex, at (A) Flexible Alignment condition, (B) 298.15 K and (C) 310.15 K using NPA/NHA/BER algorithm.

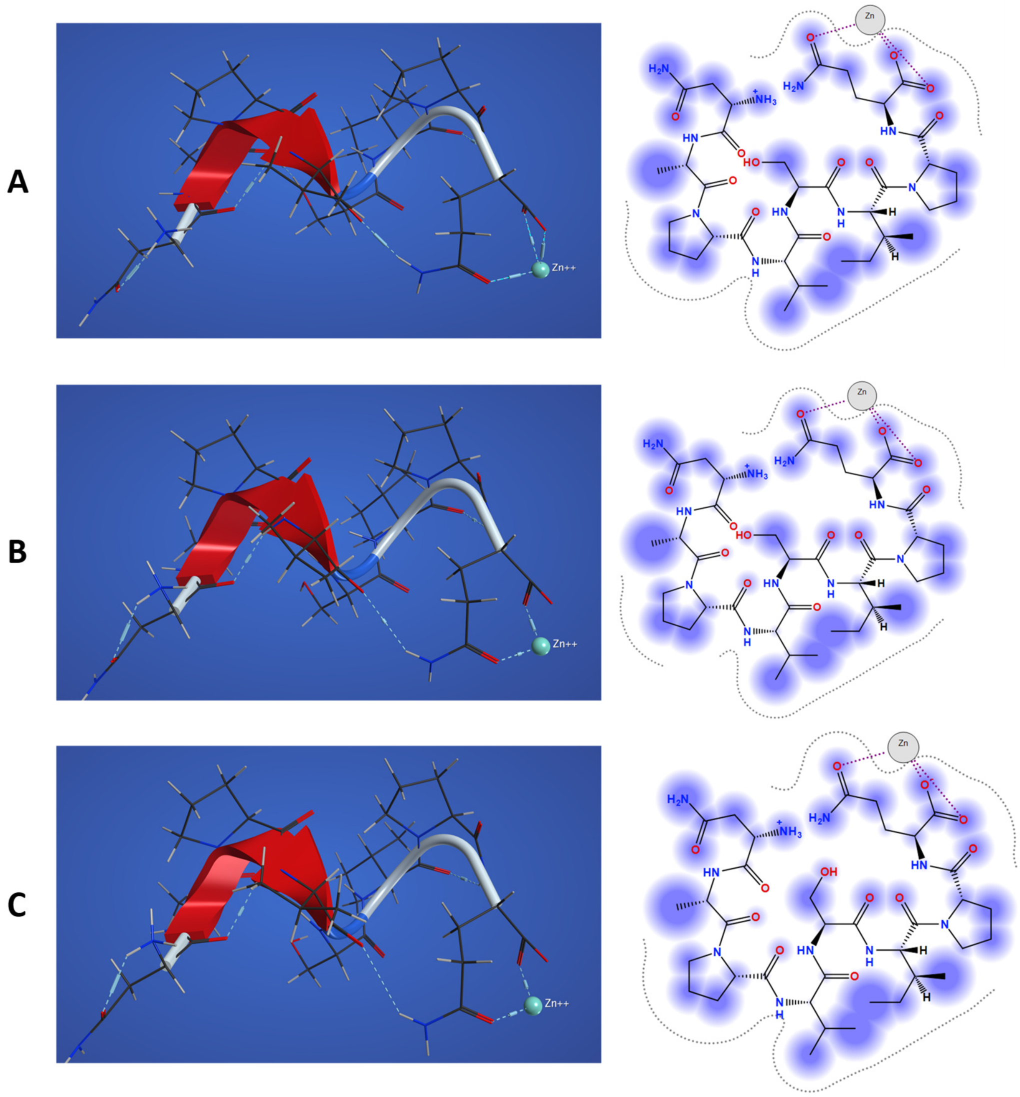

Figure S3. Molecular dynamics of NAP-Zn^{2+}-Gln^8 complex, at (A) Flexible Alignment condition, (B) 298.15 K and (C) 310.15 K using NPA/NHA/BER algorithm.

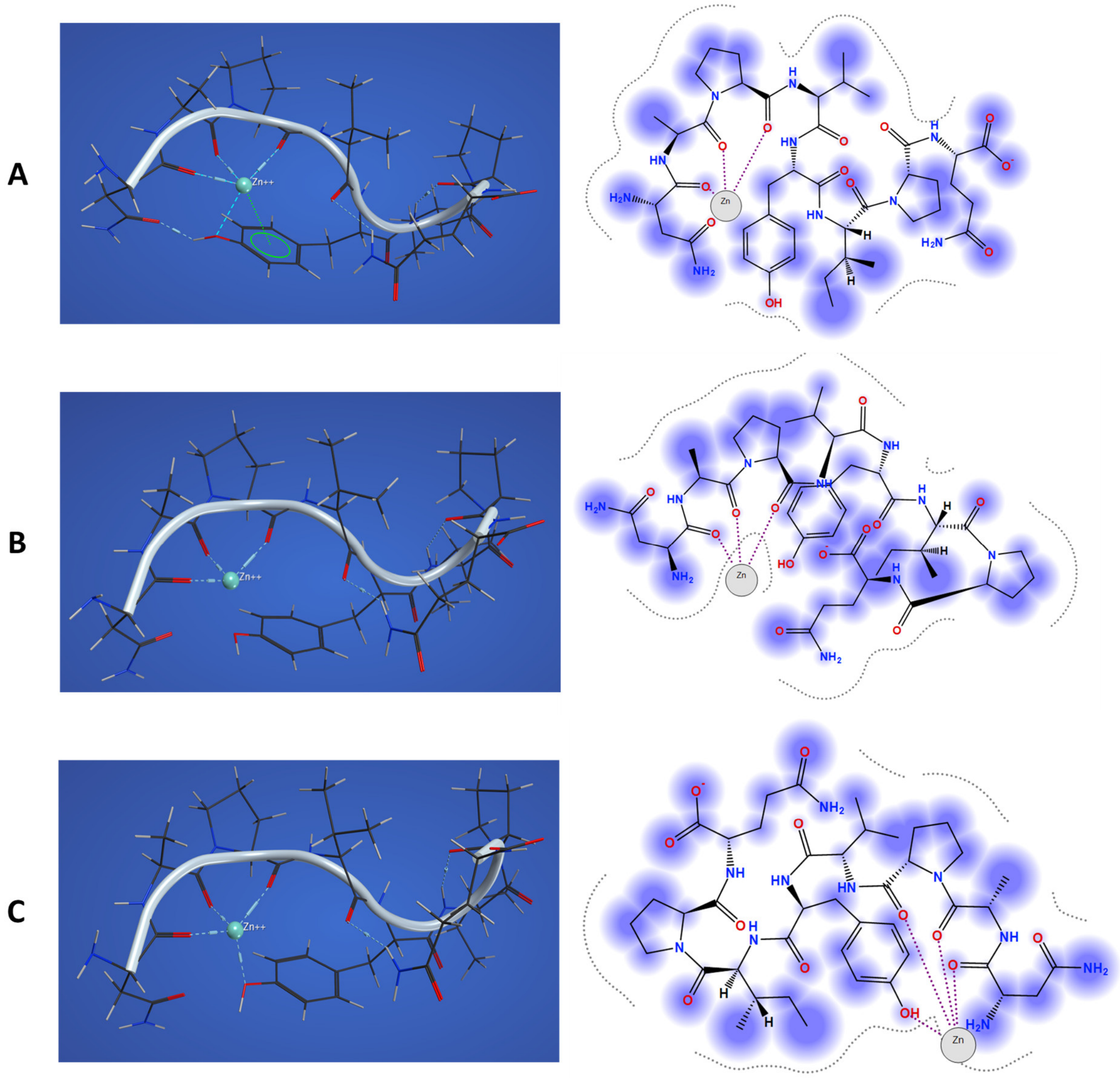

Figure S4. Molecular dynamics of NAPY-Zn^{2+} complex, at (A) Flexible Alignment condition, (B) 298.15 K and (C) 310.15 K using NPA/NHA/BER algorithm.

Table S1. The molecular parameters of NAP/NAPY-Zn^{2+} complex according to the MD simulation with NPA, NHA and BER algorithms.

Complex	Receptor	Interaction type	Distance (Å) NPA/NHA/BER	Energy (kcal/mol) NPA/NHA/BER
NAP-Zn^{2+}Asn[1](0)	O^{sp2}	Metal	1.97	-4.7
NAP-Zn^{2+}Asn[1](298.15K)	O^{sp2}	Metal	1.72<br>1.74<br>1.68	-4.0<br>-4.6<br>-4.4
NAP-Zn^{2+}Asn[1](310.15K)	O^{sp2}	Metal	1.76<br>1.76<br>1.71	-4.2<br>-4.2<br>-4.7
NAP-Zn^{2+}Gln[8](0)	O^{sp2} (acid)	Metal	2.41	-1.9
	O^{sp3} (acid)	Metal	1.85	-4.2
	O^{sp2}	Metal	2.06	-2.9
	O^{sp2} (acid)	Ionic	2.41	-9.9
	O^{sp3} (acid)	Ionic	1.85	-19.4
NAP-Zn^{2+}Gln[8](298.15K)	O^{sp2} (acid)	Metal	1.76<br>1.74<br>1.75	-3.1<br>-3.3<br>-3.6
	O^{sp3} (acid)	Metal	1.69<br>1.69<br>1.68	-3.1<br>-3.1<br>-3.5
	O^{sp2}	Metal	1.82<br>1.74<br>1.78	-2.7<br>-2.8<br>-2.3
	O^{sp2} (acid)	Ionic	1.76<br>1.74<br>1.75	-21.6<br>-22.0<br>-21.6
	O^{sp3} (acid)	Ionic	1.69<br>1.69<br>1.68	-21.4<br>-23.2<br>-21.5
NAP-Zn^{2+}Gln[8](310.15K)	O^{sp2} (acid)	Metal	1.73<br>1.76<br>1.76	-3.8<br>-3.6<br>-3.5
	O^{sp3} (acid)	Metal	1.74<br>1.69<br>1.67	-3.6<br>-3.4<br>-3.6
	O^{sp2}	Metal	1.76<br>1.76<br>1.84	-3.0<br>-2.5<br>-2.7
	O^{sp2} (acid)	Ionic	1.73<br>1.76<br>1.76	-22.3<br>-21.5<br>-21.5
	O^{sp3} (acid)	Ionic	1.74<br>1.69<br>1.67	-22.0<br>-23.2<br>-24.0

NAPY-Zn^{2+} (0)	O^{sp2} (Asn^1)	Metal	2.11	-2.8
	O^{sp2} (Ala^2)	Metal	2.12	-1.7
	O^{sp2} (Pro^3)	Metal	2.13	-3.1
NAPY-Zn^{2+} (298.15K)	O^{sp2} (Asn^1)	Metal	1.84<br>1.82<br>1.80	-2.4<br>-2.5<br>-2.6
	O^{sp2} (Ala^2)	Metal	1.83<br>1.82<br>1.90	-1.8<br>-1.5<br>-1.6
	O^{sp2} (Pro^3)	Metal	1.90<br>1.83<br>1.86	-3.8<br>-3.5<br>-3.6
NAPY-Zn^{2+} (310.15K)	O^{sp2} (Asn^1)	Metal	1.89<br>1.95<br>1.90	-2.6<br>-2.6<br>-2.4
	O^{sp2} (Ala^2)	Metal	1.79<br>1.81<br>1.84	-1.8<br>-1.8<br>-1.6
	O^{sp2} (Pro^3)	Metal	1.82<br>1.88<br>1.90	-3.5<br>-3.7<br>-3.6

where: NPA (Nosé-Poincaré-Andersen), NHA (Nosé-Hoover-Andersen), BER (Berendsen equations) and acid – the carboxyl group of glutamine.
